# How much samples do you misdiagnosis with nasal swab?

**DOI:** 10.1016/j.bjid.2024.103854

**Published:** 2024-07-20

**Authors:** Klinger Soares Faico-Filho, Ana Helena Sita Perosa, Nancy Bellei

**Affiliations:** Universidade Federal de São Paulo (UNIFESP), Escola Paulista de Medicina (EPM), Laboratório de Virologia, Departamento de Medicina, Divisão de Doenças Infecciosas, São Paulo, SP, Brazil

Dear Editor,

Timely and accurate diagnostic tests for SARS-CoV-2 are critical in the fight against the pandemic. The introduction of point-of-care tests has increased diagnostic capillarity, especially with the introduction of self-tests, in which the patient collects a nasal sample, performs the test, and interprets the results.^1^

Although samples collected from the anterior nasal cavity are less invasive and can improve adherence and test frequency, there are some issues with the performance of nasal versus nasopharyngeal collection besides its impact on the sensitivity of the self-test.^2^

This study aims to clarify whether there is a significant difference between nasal and nasopharyngeal collection methods for PCR testing of SARS-CoV-2.

This prospective study was conducted at Hospital São Paulo in São Paulo, Brazil. This study has been approved by the Ethics Committee, CAAE: 31085320.5.0000.5505. We included HCW patients who were attended the university health service. Patients should present at least 24 hours of nonspecific respiratory symptoms.

A single-team technician was trained to perform paired swabs collection on the patients. Initially, nasal swab samples were collected immediately followed by nasopharyngeal swab samples. The samples were sent to the Virology Laboratory for reverse transcriptase polymerase chain reaction (RT-PCR) analysis. The PCR test was performed according to the instructions for use of the GeneFinder™ COVID-19 Plus RealAmp Kit (OSANG Healthcare Co., Ltd.), targeting RNA-dependent RNA polymerase, envelope, and nucleocapsid SARS-CoV-2 genes, as shown here.^3^

To assess differences between patient subgroups, continuous variables were expressed by median (and interquartile range) values and compared using the Mann-Whitney *U* test. A receiver operating characteristic curve was plotted, and the area under the curve and 95 % Confidence Interval were evaluated. All analyses were performed using the SPSS 26 program. Differences were considered statistically significant at *p* < 0.05.

From September 2021 to April 2022, 195 patients were included in the study. The median age of the patients was 43 (18‒72), and 53 % was males. Each participant had paired nasal and nasopharyngeal swabs and the median number of days with symptoms was 4 (1–14). Of the samples analyzed, 10.2 % (20) were delta and 89.8 % (175) were omicron variants.

Of the 195 patients diagnosed with COVID-19 using the nasopharyngeal samples, 170 nasal samples tested positive, with a sensitivity of 88.2 % and a specificity of 100 % for these specimens collected. The Ct values obtained did not differ between the nasal and nasopharyngeal samples among participants with SARS-CoV-2 detected in both specimens (median [IQR] Ct: nasal, 23 [17.5–30] versus nasopharyngeal, 20.5 [16–30]; *p* = 0.068) (Fig. 1).

**Fig. 1:**
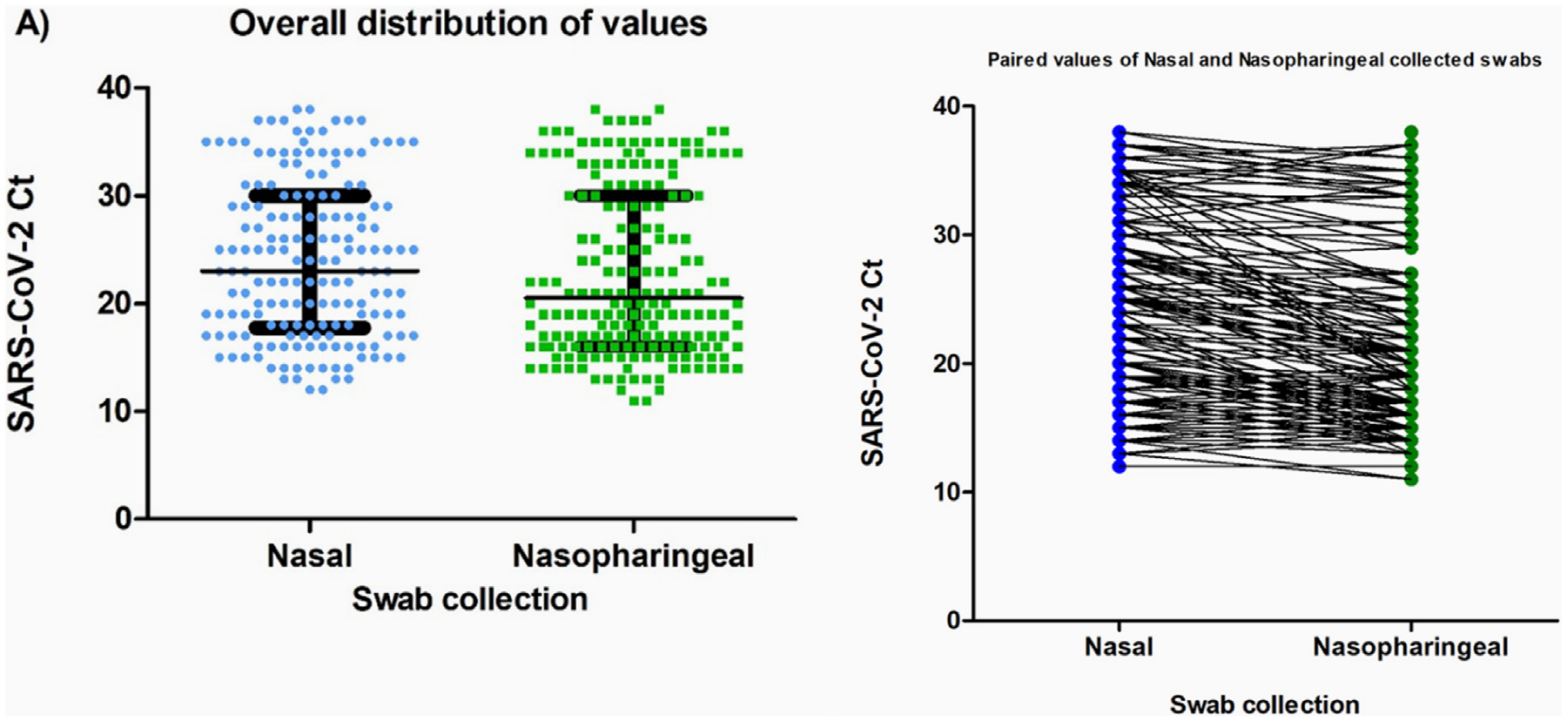
Comparison of Cycle threshold (Ct-values) according to the type of collection (nasal and nasopharyngeal). (A) General distribution of the Ct-values; the bars indicate the median and interquartile range. The Ct-values were compared using the Mann-Whitney test, with *p* = 0.068. (B) Paired Ct values according to the type of collection. All the results were obtained using RT-PCR test. Ct-values are defined as the RT-PCR cycle in which an amplification curve crosses the defined signal threshold. This is a positive result, with lower Ct-values indicating higher concentrations of viral RNA in the sample.

Fig. 2 shows the Receiver Operating Characteristic (ROC) curve of the nasal swab samples results compared to that of the nasopharyngeal swab samples (gold standard). The curve shows that the nasal swab is highly sensitive. ROC curve forecast for the nasal swab positivity result based on the nasopharyngeal swab; area under the curve result: 0.970 (0.937–1.00), *p* < 0.001.

**Fig. 2:**
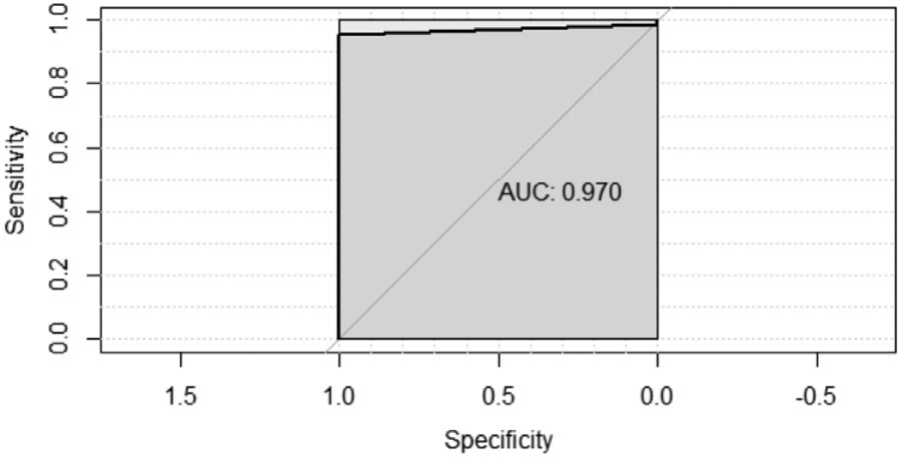
ROC curve: Comparison between the nasal and nasopharyngeal tests. AUC, Area Under the Curve.

Throughout the COVID-19 pandemic, a multitude of diagnostic test possibilities were investigated using samples of saliva, nasal secretions, nasopharyngeal or pharyngeal, and even feces specimens; the nasopharyngeal specimen swab collection was established as the gold standard.^4^

Our results show that the nasal swab has a good sensitivity (88.2 %) compared to the nasopharyngeal swab and there are no statistically significant differences in viral load between the two modes of collection. A recent antigen study with children and adolescents aged 4–14 years found no statistical difference between self-collection and collection by health professionals.^5^

Our work has some limitations. A well-trained technician performed all collections; therefore, variations can be observed when the patient collects himself. The study only compared the performance of samples in RT-PCR tests. According to WHO rapid antigen tests should meet the minimum requirements of 80 % sensitivity. When extrapolating to antigen tests, other variables, such as the sensitivity of point-of-care tests, must be considered, since at least 11.8 % may be false negative as we documented in our research.

The most important contribution of this study is that nasal collection can now be used, mainly to monitor for the presence of SARS-CoV-2,^6^ which will increase the availability of tests to a larger number of people in a more practical and cost-effective manner. It is time to use the self-test with nasal collection. However, the following maxim prevails: a negative result does not rule out the presence of the disease. The best way to control and monitor the disease is to select the best test, at the best collection time, with the best diagnostic interpretation.

## CRediT authorship contribution statement

**Klinger Soares Faico-Filho:** Conceptualization, Investigation, Writing – original draft, Writing – review & editing. **Ana Helena Sita Perosa:** Conceptualization, Investigation, Writing – original draft, Writing – review & editing. **Nancy Bellei:** Conceptualization, Investigation, Writing – original draft, Writing – review & editing.

## Conflicts of interest

The authors declare no conflicts of interest.
